# Post-Exercise Ankle–Brachial Index Is Reduced in Healthy, Young Individuals at a Level Indicating Peripheral Artery Disease

**DOI:** 10.3390/clinpract13020049

**Published:** 2023-04-17

**Authors:** Karoline Holsen Kyte, Cecilie Lunde, Jonny Hisdal

**Affiliations:** 1 Institute of Clinical Medicine, Faculty of Medicine, University of Oslo, 0318 Oslo, Norway; k.h.kyte@studmed.uio.no (K.H.K.); cecilie-lunde@hotmail.com (C.L.); 2 Section of Vascular Investigations, Oslo University Hospital, Aker, 0586 Oslo, Norway

**Keywords:** ankle–brachial index, post-exercise ABI, peripheral-artery disease

## Abstract

In young patients referred for exercise-induced pain in the legs, false positive tests are a potential problem for the post-exercise ankle–brachial index (ABI) test when using the current American Heart Association guidelines for diagnosing peripheral artery disease (PAD). The present study aimed to investigate post-exercise ABI in healthy young people, and to explore whether the current diagnostic criteria for pathological ABI should be revised. Forty-eight volunteers (18–30 years) were included. Resting examinations included ABI and ultrasound of the external iliac artery. Post-exercise examinations after a treadmill load included ABI and ultrasound of the external iliac artery; after 0 min and after 3 min. A total of 60.5% of the participants had a post-exercise decrease in ABI > 20%. A total of 6.5% showed a decrease in ankle systolic blood pressure >30 mmHg. No significant association was observed between a change in blood flow in the external iliac artery and a reduction in ABI post-exercise. Analyses of the ultrasound recordings showed no turbulence in the external iliac artery. According to the results, a 20% decrease in ABI post-exercise seems to be a physiological condition present in young people. We support the need for a reassessment of the criteria for diagnosing PAD.

## 1. Introduction

Ankle–brachial index (ABI) at rest and post-exercise is an important diagnostic tool in the assessment of patients with symptoms of peripheral artery disease (PAD). PAD is a major cardiovascular disease, characterized by atherosclerotic occlusion of arteries in the lower extremities. The prevalence of PAD is increasing worldwide and is strongly age-related [1]. Resting ABI ≤ 0.9 is indicating an obstruction of arterial blood inflow to the lower limb and is one of the criteria in diagnosing PAD [2,3].

If PAD is suspected, the resting ankle–brachial index is the preferred first test. Resting ABI is considered a specific method, but it has been criticized for not being sensitive enough, especially in patients with noncompressible vessels or less severe stenosis [4,5]. For patients with short or minor obstructions, for example, adolescents with endofibrosis or others with short isolated stenoses, resting ABI may be normal, as the stenosis has no hemodynamic significance at rest, but only when the blood flow increases [6]. Among these patients, an exercise test is recommended, according to the “American Heart Association” (AHA) recommendations. AHA further recommends using one of the two following criteria to confirm a suspicion of PAD in a patient presenting symptoms of limb ischemia; (1) a decrease in ABI > 20% post-exercise, or (2) a post-exercise ankle pressure decrease of >30 mmHg [3].

Experience from clinical practice in vascular surgery departments displays referral of young people with leg pain and suspected compromised arterial circulation. The majority of these patients show normal resting ABI values, while the post-exercise test detects a decrease in ABI > 20% in a significant proportion, consequently leading to extensive examination, including ultrasound imaging and CT/MR-angiography, without any arterial obstruction being detected [7]. Examining healthy young adults due to false positive tests may cause unnecessary risk and concern among patients, in addition to large costs for the health care system.

Earlier research has shown a 20–30% decreased post-exercise ABI in both patients with PAD, as well as in healthy and well-trained subjects [8,9]. This is most commonly explained by physiological shunting mechanisms and expanded capacity of the intramuscular artery bed during activity [10]. Further, the ABI changes are highly dependent on age, showing a significantly smaller reduction in post-exercise ABI in healthy elderly compared to the youngest [9,11]. However, there are few published results on what is considered normal ABI after treadmill load in young healthy people, and hence, there is a shortage of normal reference material for this age group [3,12,13].

Due to the lack of reference values for ABI post-exercise in healthy young people, the aim of this study was to investigate post-exercise ABI in healthy young people and to explore whether the current diagnostic criteria for pathological ABI should be revised.

## 2. Materials and Methods

The study was approved by the Regional Committees for Medical and Health Research Ethics (REC South East, reference 2018/1247). After written informed consent, 48 young volunteers between 18–30 years (32 women and 16 men) were included in the study. The participants were healthy, without any exercise-related pain in the lower limbs. No regular medication, except oral contraceptives and antihistamines, was accepted. Prior to testing, a questionnaire was given to each participant to collect information about age, height, weight, and amount of exercise per week.

After 10 min of supine resting, brachial systolic and diastolic pressure was measured in the left arm, followed by measurement of brachial and ankle systolic blood pressure using Macrolab (Macrolab, Stranden, Ålesund, Norway). Brachial systolic blood pressure was measured in both arms, while ankle systolic pressure was measured in the left ankle. During the whole experiment, participants’ heart rate was continuously monitored using a sports watch and a heart rate transmitter (Polar V800).

Ultrasound examination of flow, speed, and turbulence, distally in the external iliac artery was performed using a 9 MHz linear transducer connected to an ultrasound machine (Vivid E-95, GE Vingmed Ultrasound, Horten, Norway).

After the resting examinations, the exercise test started. The exercise test was performed on a treadmill. After a 5 min warm-up period at 10 km/h and 0% incline, a standardized ramp protocol with constant speed at 10 km/h was performed, with a 2% increase in incline every minute until a maximal incline of 20%. Participants were instructed to run for as long as possible, or to a maximum of 15 min, consistent with a maximal incline of 20%. Peak heart rate, duration of the test, and subjective experience of fatigue with the Borg Scale [14] were registered at the end of the exercise test.

When the exercise test was completed, the participants were transferred to a bed, where the ankle and brachial pressure measurements were taken after 0 min, and 3 min post-exercise. The post-exercise measurements of systolic ankle and brachial blood pressure were made in the following order: ankle first, and then the arm. The same operator performed all pressure measurements. At the same time, a second operator performed triplex ultrasound distally in the external iliac artery. Blood flow was recorded consecutively for 3 min and analyzed using the integrated software on the Vivid E95 ultrasound machine, as follows: At each point of measurement, the mean of five consecutive cardiac cycles was used to determine blood flow. In addition, while reviewing the recordings, the flow profile was visually analyzed for turbulence in the external iliac artery.

Statistical analyses were performed in SigmaPlot 14.0 (Systat Software Inc. San Jose, CA, USA). Demographical data and results are expressed as mean and standard deviation (SD). Spearman’s correlation coefficient is used for analyzing correlations between continuous variables.

## 3. Results

### 3.1. Participants

The characteristics of the study group are presented in Table 1. All participants were healthy and did not have claudication symptoms or other symptoms of PAD.

### 3.2. Resting Values

Results from the pre-exercise measurements are presented in Table 2. Resting ABI was within the normal range in all participants (0.9–1.4) with an average of 1.13 (±0.10) [3].

### 3.3. Post-Exercise Results

The post-exercise results are presented in Table 3. The mean reduction in ABI post-exercise was 22.9% (±12.0), and the mean post-exercise absolute change in systolic ankle pressure was an increase of 12.2 mmHg (±24.5), both representing a significant difference compared to resting values (*p* < 0.01). The 3 min post-exercise ABI was 0.95 (±0.13), and the mean recovery of the ABI to baseline value within the first 3 min was 84%. For all participants, the mean peak heart rate was 194 (8.0) and the duration of the test was 12.3 (2.2) min. The Borg scale (6–20) at the end of the test was 17.6 (1.4).

**Figure 1:**
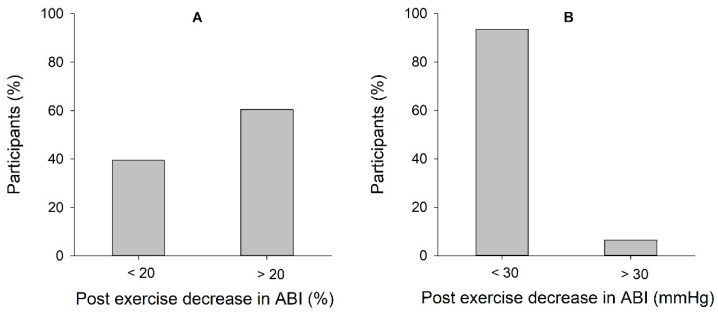
Participants’ test results, divided into pathological and non-pathological results according to the two different AHA recommendations. (**A**) Shows the distribution according to the criteria of a decrease in ABI > 20% post-exercise. (**B**) Shows the distribution according to the criteria of a post-exercise ankle pressure decrease of >30 mmHg. “Post exercise” refers to 0 min post-exercise.

Twenty-nine participants, equivalent to 60.5%, had a post-exercise decrease in ABI > 20% (Figure 1A). Nineteen participants, 39.5%, had a post-exercise decrease in ABI < 20%. Three of the participants, 6.5%, showed a decrease in ankle systolic blood pressure >30 mmHg, while the rest of the participants, 93.5%, had a decrease in ankle systolic blood pressure <30 mmHg (Figure 1B). The post-exercise decrease in ABI for the three participants with a decrease in ankle systolic blood pressure >30 mmHg, were all >35%.

**Figure 2:**
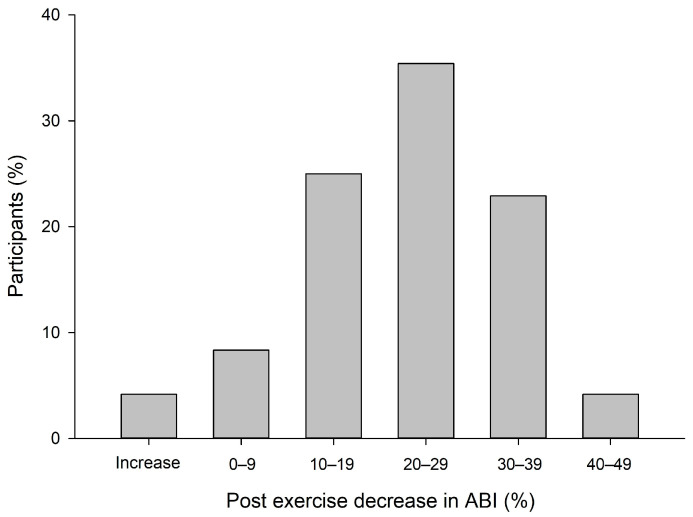
The distribution of participants, subdivided into groups of % reduction in ABI post-exercise. “Post exercise” refers to 0 min post-exercise.

Figure 2 shows the distribution of participants, subdivided into groups of % reduction in ABI post-exercise. Most of the participants distribute between a 10–40% decrease in ABI. Further, two participants had an increase in post-exercise ABI, while no participants had a decrease >50% post-exercise.

No significant association was observed between a change in blood flow in the external iliac artery and a reduction in ABI post-exercise (r^2^ = 0.12). Analyses of the ultrasound recordings showed no turbulence in the external iliac artery in any of the participants.

## 4. Discussion

In the present study, 60.5% of the participants showed a pathological decrease in ABI post-exercise, according to one of the AHA’s criteria for PAD (>20% decrease in ABI post-exercise) [3]. All participants were healthy, without any exercise-related pain in the lower limbs, and with no observed turbulence in the external iliac artery post-exercise. This explains, and underlies, the problem of several false positive tests, using post-exercise ABI in the diagnosis of PAD in young patients, which further leads to unnecessary comprehensive investigation, concern among patients, and large costs for the health care system.

AHA recommends two different criteria for diagnosing PAD when resting ABI is within normal reference values. Interestingly, we experienced a large discrepancy between the two criteria when analyzing our results. In the present study, the mean reduction in ABI post-exercise was 22.9% (±12.0), which fulfills the AHA criterion to diagnose a patient with peripheral artery disease if the person suffers from simultaneous pain in the lower limbs. The mean post-exercise absolute change in systolic ankle pressure was 12.2 mmHg, which is not considered pathological according to the AHA criterion. Explained in a different way, in the present study, 60.5% fulfilled the AHA criterion of >20% decrease in ABI post-exercise, while only three participants (6.5%) met the AHA criterion of >30 mmHg reduction in ankle systolic blood pressure post-exercise. There were no participants with a reduction of >30 mmHg among the participants who did not fulfill the AHA criterion of >20% decrease in ABI post-exercise. The criterion of >30 mmHg reduction in ankle systolic blood pressure must be considered very specific, but probably less sensitive. In contrast, the criterion of >20% decrease in ABI has a low specificity and a high sensitivity, with the consequence of investigating several healthy people with PAD and providing several false positive results.

High specificity and sensitivity are both essential features of medical tests, and it may appear that the two different post-exercise criteria for diagnosing PAD each represent their extreme edge in terms of sensitivity and specificity. The fact that two established criteria for the same condition show such a large discrepancy, supports the need for a reassessment of the criteria and reference material for young individuals presenting with leg pain and suspected compromised arterial circulation.

The three participants in the present study, who fulfilled the AHA criterion of a decrease in ankle systolic blood pressure >30 mmHg, all had a decrease in ABI > 35% post-exercise. This substantiates that using the criterion of >30 mmHg reduction in ankle systolic blood pressure will increase the specificity in diagnosing PAD and supports that the AHA criterion of >20% decrease in ABI post-exercise includes too many false positives. Further illustrating the large discrepancy between the two AHA criteria.

Our results are thus consistent with the findings of Mahe et al. [12], who showed a great discrepancy between the two AHA criteria used for post-exercise ABI. In the study of Mahe et al., one of five patients was classified differently according to the criteria, where a decrease of >30 mmHg in systolic ankle pressure was considered a stricter criterion than a relative change in ABI of >20%.

Further, the criterion of a reduction of >30 mmHg in ankle systolic blood pressure is based on a study of young adults [7]. This illustrates that a stricter criterion for PAD may be suitable in a younger population, like in our study, while it may not be sensitive enough in an older patient group. Aging induces changes in the arterial wall, modifying the pressure and the ABI [11], thus explaining the importance of age-matching when establishing reference material for ABI.

Moreover, the criterion of >20% decrease in ABI post-exercise is based on a study by Ouriel et al., where they found a significantly higher decrease in ABI (40 ± 25%) among the PAD patients compared to the control group (7 ± 7%) [13]. Interestingly, the healthy control group in the study of Ouriel et al., which forms the basis for today’s criteria for pathological ABI post-exercise, were all younger than 30 years. This is in line with the population making the basis for the 30 mmHg criteria [7], but still, the discrepancy between these criteria has been shown in several studies and seems to be a real problem in the clinic. Taking into consideration the large gap between a 7 ± 7% decrease in healthy, control limbs versus approximately 40 ± 25% in PAD patients, the criterion of a post-exercise decrease in ABI > 20% seems to be made on a slightly insufficient basis and supports the need for a new reference material for ABI post-exercise.

Mahe et al. further rises a critical question of whether we can keep the results from Ouriel et al. to define post-exercise criteria for PAD in a position paper [12]. Based on our findings and earlier research as discussed above, we are in agreement with Mahe et al. and support the need for a renewal of the criteria.

In our study, all participants were healthy and did not have claudication symptoms. We did not observe signs of stenosis or turbulence in the external iliac artery in any of the participants before or after exercise. This indicates that the decrease in ABI post-exercise is not due to reduced blood inflow to the lower extremities, and, therefore, must have another explanation. This is in contrast to what Desvaux et al. suggested in their study, that the decrease in ABI may be due to turbulence at higher flow levels rather than shunting of blood to active muscles [15]. Our results with normal blood flow in the external iliac artery in all participants make this doubtfully, pointing to a shunting mechanism as an alternative explanation of the pressure decrease, where blood is shunted away from the foot to the working muscles. In addition, we found no association between a change in flow in the external iliac artery and a fall in ABI, which supports the theory that pathological conditions in the external iliac artery do not explain the decrease in ABI.

Another alternative hypothesis explaining why the ABI is reduced after exercise in individuals without pathological conditions in arteries is the role of the endothelium. Physical activity influences the endothelial layer by stimulating the production and release of several substances causing vasodilation. Among multiple substances, we will highlight nitric oxide (NO), prostacyclins, and endothelium-derived hyperpolarizing factor (EDHF) as important modulators. These substances are released in increased amounts from the endothelium under conditions of increased shear stress and induce endothelium-dependent vasodilatation [16,17].

From clinical experience at the vascular surgery department, where young patients with pathological conditions in arteries of the lower limbs are referred, we have observed a remarkable turbulent flow in most of the patients, which further explains the decrease in ABI post-exercise. These patients often have normal resting ABI, as the stenosis has no hemodynamic significance at rest, but only when the blood flow increases. The blood flow becomes turbulent, and a decrease in pressure occurs, when the blood flow reaches critical velocity, either due to stenosis or incredibly large blood flow to the legs. In clinical practice, we have observed a decrease in ABI of nearly 80% in these patients, with a simultaneous paling of the symptomatic leg. The lack of turbulence among the healthy young participants in the present study increases the probability that the participants who fulfilled the AHA’s criterion for PAD are false positives. The combination of ultrasound imaging and pressure measurements post-exercise may therefore increase the specificity for diagnosing pathological conditions in the arteries of the lower limbs.

Another interesting finding in the present study is the percentage of participants recovering to baseline values of ABI within the first 3 min. It is earlier reported in a statement from the American Heart Association [3] that “A recovery of at least 90% of the ABI to baseline value within the first 3 min after exercise was found to have a specificity of 94% to rule out PAD”. The participants in our study recovered 84% within the three first minutes. However, it is previously shown that the recovery time of the ABI correlates well with the intensity of the exercise protocol. This must be considered when evaluating recovery time. Our protocol led to exhaustion for the majority of the participants, with a mean Borg scale score of 17.6, defined as “very hard” [14]. Hence, this may explain why our participants did not reach 90% recovery of the resting ABI after 3 min. Accordingly, we agree that recovery time might be useful as a supplement in diagnosing PAD, provided that the protocol used is taken into consideration.

The present study is not without limitations. Post-exercise measurements were performed unilaterally, and, therefore, any intraindividual side differences are not taken into consideration. For ankle pressure measurements, we exclusively used arteria tibialis posterior, although the ABI is based on the highest pressure in the current foot. We cannot exclude that some of the participants had higher pressure in arteria dorsalis pedis than in arteria tibialis posterior. Due to the lack of reference values for ABI post-exercise in healthy young people, it was not possible to perform a valid power calculation prior to the study. However, the results revealed differences, indicating statistical power sufficient to demonstrate a significant fall in ABI post-exercise in the majority of the test subjects.

In all participants, post-exercise ankle pressure was measured before arm pressure. This gives time for the arm pressure to slightly decrease compared to the ankle pressure after exercise, consequently underestimating the decrease in ABI. However, this makes it even more difficult to meet the criteria for peripheral artery disease.

Finally, the test protocol may also affect the results. In previous studies, a number of different protocols have been used, and significant differences have been found in ABI after maximum exercise in cycle ergometers and treadmill [18]. We decided to use a time-efficient ramp protocol, which allows a quite far and steep run at the same time, leading to exhaustion for the vast majority before the test was completed. Still, we cannot guarantee that this is the most appropriate protocol.

## 5. Conclusions

In conclusion, a 20% decrease in ABI post-exercise seems to be a physiological condition present in healthy young individuals. We support the need for a reassessment of the criteria for diagnosing PAD. A combination of ultrasound imaging and pressure measurements post-exercise may be used for increasing the specificity and sensitivity for diagnosing pathological conditions in the arteries of the lower limbs. The present study is small, and there is a need for further studies with a larger sample size for establishing recommendations and reference material for young individuals. This is important to support the investigation when suspecting compromised arterial circulation.

## Figures and Tables

**Table 1:** Participants’ characteristics.

Variable	Females (n = 32)	Males (n = 16)	Total (n = 48)	*p*-Value
Age	23.2 (2.6)	24.4 (2.7)	23.6 (2.6)	0.17
Height	167.7 (5.5)	179.8 (6.5)	171.7 (8.2)	<0.05
Weight	61.0 (6.1)	77.2 (8.6)	66.4 (10.3)	<0.05
Amount of exercise (hours/week)	4.8 (2.4)	6.0 (2.7)	5.2 (2.6)	0.14

Values are mean (SD). *p*-values refer to differences between females and males.

**Table 2:** Resting values for study group.

Variable	Females (n = 32)	Males (n = 16)	Total (n = 48)	*p*-Value
Heart rate (beats/min)	67 (12.6)	69 (8.6)	68 (11.0)	0.66
Arm systolic blood pressure (mmHg)	110 (9.3)	125 (10.6)	115 (12.0)	<0.05
Arm diastolic blood pressure (mmHg)	70 (7.4)	73 (7.0)	71 (7.0)	0.12
ABI	1.14 (0.1)	1.10 (0.1)	1.13 (0.1)	0.20
External iliac artery flow (mL/min)	520 (238.6)	743 (396.5)	594 (315.0)	0.05
PSV (m/s)	0.99 (0.21)	0.99 (0.14)	0.99 (0.19)	0.92

Values are mean (SD). ABI = ankle–brachial index. PSV = peak systolic velocity. *p*-values refer to differences between females and males.

**Table 3:** Post-exercise values for study group.

Variable	0 min	3 min
Post-exercise ABI	0.87 (0.16)	0.95 (0.13)
Decrease in ABI (%)	22.9 (12.0)	15.6 (11.9)
Absolute change in systolic ankle pressure (mmHg)	+12.2 (24.5)	−18.6 (14.7)
PSV (m/s)	1.94 (0.3)	1.47 (0.3)
Post-exercise ABI in % of baseline ABI	77 (12.0)	84 (12.0)
Post-exercise external iliac artery flow (mL/min)	2429 (741.0)	1733 (534.0)

Values are mean (SD). ABI = ankle–brachial index. PSV = peak systolic velocity.

## Data Availability

The data present in this study are openly available in the Supplementary Materials as S1.

## Supplementary Materials

The following supporting information can be downloaded at: https://www.mdpi.com/article/10.3390/clinpract13020049/s1, S1: Raw data. CSV file containing the raw data from the research.
